# The Mosquito and Malaria

**Published:** 1902-12

**Authors:** A. N. Perkins

**Affiliations:** Sabine Pass, Texas


For Texas Medical Journal.

### The Mosquito and Malaria.

BY A. N. PERKINS, M. D., SABINE PASS, TEXAS.

The theory that the mosquito is the principal factor in imparting malaria to the human family has been enthusiastically accepted by some intelligent members of the medical profession. They believe the theory has been practically demonstrated and are inclined to consider those who refuse to accept it as true as old fogies similar to those who protested against Harvey's theory of the circulation.

I do not believe the theory is correct, and will briefly give a few of my reasons for entertaining this opinion.

In the first place, I would say the organs of the mosquito are not suited to perform such a function. The mosquito lights on his victim and aspirates—that is, sucks from his victim blood with which he fills himself. He injects nothing, nor can he do it. The rattlesnake inflicts a wound and injects into it a deadly poison. The mosquito has no organ by which he can inject. He can only aspirate—that is, suck the blood from his victim.

In the second place, my experience and observation in a mosquito country give me no facts corroborative of the theory. The coast country of this State is a paradise for the mosquito. They are, at times, as innumerable as the sands of the desert. They obstruct the rays of the sun and cast shadows upon the moon. The cattle that feed upon the prairie are often destroyed by them. But, notwithstanding all this, the coast country of Texas is the most healthy and the most free of malarial diseases of any part of the State. I

know families who have lived in this part of the State for more than twenty years who have never had a chill nor taken a dose of quinine. How can such an extraordinary immunity from malarial diseases be accounted for if mosquitoes are the chief factors in imparting malaria to man?

No one despises this devilish insect more than I do; no one would more gladly see them exterminated from the face of the earth, but their crimes are great enough without accusing them of crimes of which they are innocent. "Let justice be done even to the mosquito."

